# Predictive Role of Body Image in Bulimic Behaviors Among Obese Patients Qualified for Bariatric Surgery

**DOI:** 10.3389/fpsyt.2021.781323

**Published:** 2021-11-25

**Authors:** Barbara Bȩtkowska-Korpała, Aleksandra Ćwiȩk, Bernadetta Izydorczyk, Anna Starowicz-Filip, Piotr Major

**Affiliations:** ^1^Department of Psychiatry, Department of Medical Psychology, Jagiellonian University Medical College, Krakow, Poland; ^2^Department of Clinical Psychology, University Hospital in Krakow, Krakow, Poland; ^3^Department of General, Oncological, Metabolic and Emergency Surgery, Centre for the Surgical Treatment of Obesity, University Hospital in Krakow, Krakow, Poland; ^4^Institute of Psychology, Jagiellonian University, Krakow, Poland; ^5^2nd Department of General Surgery, Jagiellonian University Medical College, Krakow, Poland

**Keywords:** body image, obesity, bariatric treatment, bulimic eating behavior, MBSRQ Cash, EAT-26 Garner

## Abstract

Bulimic behavior and the associated experience of one's own body are of great importance in the course of surgical treatment for obesity. This study determined the predictive role of multidimensional body image on bulimic-type eating behaviors among individuals scheduled for the surgical treatment of obesity. This study was conducted in a clinical setting on a group of 100 obese patients who were treated at the Centre for the Surgical Treatment of Obesity at the University Hospital in Krakow (Poland) and were qualified for bariatric surgery. Body image was examined with Cash's Multidimensional Body-Self Relations Questionnaire (MBSRQ) and bulimic behavior with David M. Garner's Eating Attitudes Test (EAT-26). Part A of the EAT-26 focused only on the bulimia and food preoccupation scale. Part B included sex, age, and body mass index (BMI) in the predictive model. A stepwise multiple regression analysis was conducted to assess psychological predictors of eating behavior. For binary variables, a logistic regression analysis was conducted for the whole group and for the women's group alone. Owing to the small sample size of men, regression analyses were not conducted. Higher values were observed in the Appearance Orientation dimension among women when compared to men. Appearance evaluation and age were found to be significant predictors for bulimic behaviors in the whole group. In regression models for behavior in the last 6 months, the predictors were found to be Health Evaluation and Appearance Orientation for laxative use, and Overweight Preoccupation for vomiting for weight control. Health-promoting behaviors in obesity treatment were conditioned as follows: for exercise, the predictors were sex and Fitness Orientation and for weight loss, they were Overweight Preoccupation and Body Areas Satisfaction. Our study shows that different bulimic behaviors are variously conditioned by body image dimensions, some of which are predictors of behaviors that are risk factors for obesity and poor outcomes of bariatric treatment, whereas others increase the chance of pro-health behaviors among obese individuals.

## Introduction

Obesity is a major health problem worldwide, as reflected by its steadily increasing prevalence in the adult population (1–3). A WHO report shows that 39% of the global adult population was overweight in 2016, and that 13% suffered from morbid obesity (1). A body mass index (BMI) above 30 kg/m^2^ indicates clinical obesity and its diagnosis is associated with a higher risk of numerous somatic health consequences like cardiovascular disease, hypertension, type 2 diabetes (4), and respiratory diseases (5) including asthma (6). Obesity is a risk factor for cancer (7) and also leads to disturbances in psychosocial functioning. Multiple experiences of discrimination as a result of increased body weight imply lower quality of life scores (8), reduced body satisfaction, lower self-esteem, a tendency to experience negative emotional states, and poor cognitive functioning (9).

With the increasing prevalence of obesity, more radical forms of combating the problem are gaining recognition, such as bariatric surgery, which is currently the most effective and durable treatment for morbid obesity (10). This type of surgery provides significant weight loss and helps treat over 40 obesity-related conditions (11, 12). However, a comparative analysis of the durability of bariatric treatment outcomes shows a marked variability in distant outcomes regardless of the type of surgical procedure (10, 13). It is important to identify variables with predictive value for the outcome of the surgical treatment of obesity (14).

A literature review indicates that a multiplicity of psychological factors are significant in the development of obesity, in the course of its treatment and the post-operative outcomes (15, 16).One of the most significant psychological factors among people with obesity is the presence of pathological eating patterns (eating disorders or ED), which is defined as persistent, abnormal eating behaviors over time that result in altered food intake or consumption leading to impaired physical health and/or psychosocial functioning (17). The American Psychiatric Association's classification of mental disorders (Diagnostic and Statistical Manual of Mental Disorders—DSM-5) includes a group of non-specific eating disorders in addition to anorexia nervosa (AN) and bulimia nervosa (BN). It includes, among other things, binge eating disorder (BED), which is characterized by recurrent episodes of overeating accompanied by anxiety and a feeling of lack of control over eating despite the absence of physiological hunger. Eating large amounts of food at a time, eating faster than usual, eating alone out of embarrassment, and feelings of shame and guilt or lowered mood after a binge are the characteristic features of BED. Episodes of overeating must occur at least once a week for at least 3 months (17). In contrast to bulimia nervosa, binge eating is not followed by compensatory behaviors like provoking vomiting and/or using laxatives. Co-occurrence of eating disorders, especially BED, is reported in the obese population. Over half of the people with clinically diagnosed BED have a BMI that corresponds to being overweight or obese (18). BED is highly prevalent among patients who participate in conservative obesity treatment (19) and who are qualified for bariatric treatment; it affects up to 27% of them (20). In a longitudinal study involving a 7-year follow-up of patients treated surgically for obesity, Lavender et al. (13) demonstrated that the greater the severity of an eating disorder (eating pathology), the poorer the effect of bariatric treatment. Eating behaviors with bulimic features affected ~40% of patients before bariatric surgery, and 24% of them reported overeating at least once a week (21). After the treatment, a lower severity of this type of eating behavior was observed (22–25).

Bulimic eating behavior is closely related to the experience and image of one's own body (26, 27). According to Thomas F. Cash's theoretical model of multidimensional measurement of body image, this attitude includes both the perceptual and the behavioral dimension of one's own corporeality. Body perception includes satisfaction or dissatisfaction with one's appearance, one's own health and physical condition. The behavioral dimension includes actions aimed at improving the appearance and attractiveness of one's body and its physical condition (28). It is important to identify how a person perceives their body image as it can be a predictor of compulsive and bulimic disorders (29), which, in turn, may be relevant to the treatment of obesity (22, 30–32).

Dissatisfaction with one's body and disturbances in body image perception can take many forms and occur regardless of sex, age, or ethnicity. They affect both normal weight individuals and those belonging to extreme categories in terms of BMI (underweight and morbid obesity) (33). No research model has created or used a uniform definition of body image among obese people. However, a review of the literature by Ivezaj et al. (16) shows that body image dissatisfaction among people with obesity is common and associated with various aspects of biopsychosocial functioning, including eating behavior. Individuals with a higher BMI estimate body shape more adequately than do thin and overweight individuals with bulimic behavior or symptoms of binge eating, but individuals after bariatric surgery with comorbid eating disorders overestimate the size of their bodies (16). Geller et al. (22) noted the mediating role of emotional eating between body image dissatisfaction and levels of stress experienced among individuals undergoing treatment for obesity. A clear association of body image dissatisfaction with levels of anxiety, depression, and the occurrence of suicidal thoughts was indicated (31, 32).

Eating behavior and body image appear to play a key role in the long-term effectiveness of bariatric surgery for the treatment of morbid obesity. Research on the role of psychological factors in the effectiveness of these surgeries must seek to understand both the psychological mechanisms that are important for one leading a pro-healthy lifestyle, and the subjectively assessed quality of life after surgery. For example, impaired perception of body size (34) and the association of body image dissatisfaction with levels of stress experienced because of stigmatization (35) has been reported among obese individuals seeking bariatric treatment. De Panfilis et al. indicated that improvement in body image after bariatric surgery is not related to objective treatment outcomes, which is understood as the number of kilograms reduced (30). Bariatric treatment may be associated with the lack of acceptance of excess skin and general dissatisfaction with the appearance of one's skin, which will generate persistent pre-surgical dissatisfaction with the body or particular parts of it. De Panfilis et al. indicated the importance of the modulatory role of eating disorders, including bulimic behavior, in predicting treatment outcomes (30).

In light of the problems described, we conducted this study to determine the predictive role of multidimensional body image on bulimic eating behavior in individuals scheduled for the surgical treatment of obesity. This is an important topic to address because of the role of bulimic behavior both in the development of obesity and its treatment (21, 23–25), and their determinants. The study also points out the need for the analysis of a multidimensional body image. Bertoletti et al. (36) postulated that future studies should analyse body image in the context of its functionality and impact on changes in the quality of life after bariatric surgery. Grilo et al. (37) conducted a review on body image in bariatric patients and highlighted the paucity of research on the multidimensional examination of body image and its importance in the treatment of people with obesity. We referred to the theory and psychometric measurement of multidimensional body image using the MBRSQ tool (28, 38). This tool measures both the level of satisfaction with one's body and aspects related to fitness and health. The issue analyzed has an implementation value, too. Bertoletti et al. (36) justified that treatment outcomes should be defined with the patient before surgical treatment, including the identification of body satisfaction indicators as an element related to the quality of life after surgery.

Owing to the exploratory nature of this study, research questions were formulated instead of hypotheses:

1. Are there differences between men and women in terms of multidimensional body image and bulimic behavior?
2. Are body image dimensions predictors of bulimic eating behavior among patients scheduled for bariatric surgery?

The research model assumed that the explanatory variables were body image dimensions, BMI value, sex, and age, whereas the response variables were indicators of bulimic eating behavior. The selection of the operationalizing variables presented above is consistent with the clinical and psychosocial characteristics of the study group. The results obtained attempt to answer the research questions posed. We predict that the analysis of the relationship between the multidimensional body image and bulimic behavior will have practical implications for the targeted planning of psychological interventions in this group of patients.

## Materials and Methods

### Ethics Statement

All procedures followed were in line with the ethical standards of the responsible committee on human experimentation and the Helsinki Declaration of 1975, as revised in 2000. The Bioethics Committee of the Jagiellonian University has approved the study (Document No.: 1072.6120.16.2021, dated February 17, 2021). Informed consent was obtained from all participants included in the study.

### Participants

This study was conducted between January and July 2021 at the Centre for the Surgical Treatment of Obesity at the University Hospital in Krakow. The inclusion criteria for the study group were follows: eligible for surgical treatment of obesity, primary surgery for the treatment of obesity, and the absence of a chronic, untreatable mental health disorder, which would make it difficult to maintain cooperation with the treatment team. The criteria for bariatric surgery for the treatment of morbid obesity followed the Polish Surgeons Society's Guidelines of The Section of Metabolic and Bariatric Surgery, which includes a BMI of 40 or 35 kg/m^2^ with obesity comorbidities (39).

The study group included 100 patients (73 women and 27 men) aged between 21 and 64 years (mean age 39.25 years, *SD* = 9.92). Mean BMI = 44.42 (*SD* = 6.52, min. 33, max. 63). As many as 78, 20, and 2% of the subjects had class 3, 2, and 1 obesity, respectively. The patients were examined by a clinical psychologist after a surgical consultation, which indicated the need for bariatric surgery. The study was carried out in natural clinical conditions in a clinical application as part of the qualification procedure for the surgical treatment of obesity. Following the tests, the patients waited for the date of the procedure, which was not specified during the qualification period. Patients were informed about the objectives of the study in keeping with the guidelines of the Bioethics Committee of the Jagiellonian University.

### Methods

Two tools were used in the study:

1. The Polish version of the Eating Attitudes Test (EAT-26) by Garner et al. (40) was used to study eating behavior (41). EAT-26 comprises of two parts, named A and B. The respondent assesses statements on a scale of 1–6, except for the last two questions, which are answered with “yes” or “no.” Part A has 26 items and deals with eating behaviors and feelings in the following categories: dieting, bulimia, food preoccupation, and oral control. A score of over 20 on the total scale in this section justifies a clinical interview for an eating disorder. Section B refers to the frequency of behaviors that may be symptoms of an eating disorder in the preceding 6 months, such as overeating with the feeling of not being able to stop eating (EAT-B1), vomiting to affect one's weight or shape (EAT-B2), using laxatives, diet pills, or diuretics to control one's weight or shape (EAT-B3), exercising for more than an hour a day to lose or control weight (EAT-B4), losing 10 kg or more (EAT-B5), and information on treatment for an eating disorder ever (EAT-B6). In the Polish version, the application of the EAT-26 on a non-clinical sample indicated an overall Cronbach's alpha of 0.80 (41). The Bulimia and Preoccupation with Food Scale was used in this study. The sum of Part A and the Dieting scales and Oral control scales were not interpreted because they refer to behaviors that may be indicators of psychological eating disorders of the restrictive type, but in the group of people with obesity these behaviors manifest as positive changes in eating patterns. In using the EAT-26 questionnaire, summary scores from part A and two subscales were not included because of the content of the Dieting and Oral Control scales, which represent strategies undertaken to initiate and perpetuate health-promoting changes in eating behavior in this group of patients. One example is the expectation of a weight reduction of 5–10% of initial body weight in preparation for the surgical treatment of obesity. Statements that indicate problems with anorexia or bulimia are favorable in this case, such as: “*I avoid food with sugar*,” “*I eat diet food*,” *and* “*I take steps to reduce my body weight*” in Part A of the questionnaire and an affirmative response to the questions: “*In the past 6 months, did you happen to exercise for more than 60 min a day to lose or control weight?*” *and* “*In the past 6 months, did you happen to lose 10 kg or more?*” in Part B of the questionnaire illustrate the strategies used by patients to reduce weight.
2. Rogoza et al.'s Polish adaptation (41) of the Multidimensional Body-Self Relations Questionnaire (MBSRQ) created by Cash (38) examined the assessment of the respondent's attitude toward their own bodies. In the Polish normative group, the exploratory factor analysis showed that factor structure of the MSBRQ was similar to that of the original version. Its internal reliability was assessed using the McDonald x factor and ranged from 0.66 to 0.91. The respondent referred to each of the 69 items on a scale that ranged from 1 to 5. The higher the score, the greater the satisfaction with the body areas and its functions. The test included the following scales: Appearance evaluation (AE), Appearance orientation (AO), Fitness evaluation (FE), Fitness orientation (FO), Health evaluation (HE), Health orientation (HO), Illness orientation (IO), Body areas satisfaction (BAS), Overweight preoccupation (OP), and Self-classified weight (SCW).

Medical history data included: sex (female-male), age, BMI, and comorbid psychiatric disorders.

### Statistical Methods

The results were analyzed using the SPSS 27 package (42). The first step was to present the mean values of the variables in the research model. The differences between the mean intensity of the variables in the male and female groups were measured. The Mann-Whitney *U* and *chi*^2^ tests were applied at this stage. The strength of the association between the psychological response and explanatory variables was measured using correlation analysis. The stepwise multiple regression analysis was used to estimate the strength and direction of psychological predictors of eating behavior. Logistic regression analysis was used for binary variables for the whole group and the women's group alone. Owing to the small number of men, regression analyses were not performed in this subgroup.

## Results

Table 1 presents the descriptive characteristics of the body image and eating behavior variables.

**Table 1:** Characteristics of psychological variables in the whole sample group (for continuous variables).

**Psychological variables**	**M**	***SD***	**Min–Max**
**MBSQR**			
AE—Appearance evaluation	1.90	0.72	1.00–4.00
FE—Fitness evaluation	2.19	0.79	1.00–40.0
HE—Health evaluation	2.91	0.67	1.26–4.50
AO—Appearance orientation	3.38	0.68	1.83–4.75
FO—Fitness orientation	2.77	0.52	1.33–4.08
HO—Health orientation	3.24	0.50	1.91–4.50
IO—Illness orientation	3.13	0.79	1.20–5.00
OWP—Overweight preoccupation	3.54	0.75	1.75–5.00
SCW—Self classified weight	4.74	0.63	1.00–5.00
BAS—Body areas satisfaction	2.24	0.55	1.00–3.77
**EAT-26 (bulimia and food preoccupation)**			
B—Bulimia and food preoccupation	2.10	2.77	0.00–12.0
EAT-B1 (overeating)	1.95	1.27	1.00–6.00
EAT-B2 (use of laxatives)	1.05	0.26	1.00–3.00
EAT-B3 (vomiting)	1.61	1.31	1.00–6.00
EAT-B4 (exercising more than 60 min a day)	1.79	1.23	1.00–6.00

Of those surveyed, 33% had lost more than 10 kg in the preceding 6 months and 26% had received treatment for an eating disorder in the past. To answer the first question on the differences between men and women in terms of multidimensional body image and bulimic eating behavior, the non-parametric Mann-Whitney *U*-test was used (Table 2). The choice of test was also made because of the large difference in the number of women (*n* = 73) and men (*n* = 27). In the analyses, higher values were observed only in the AO dimension among women when compared to men (AO—F: mean 3.47, *SD* = 0.67 vs. M: mean 3.11, *SD* = 0.64, *p* = 0.017).

**Table 2:** Differences between men and women in the severity of psychological variables.

**Psychological variables**	**Mean**		***SD***		***t*_**(98)**_**	***p***
	**F**	**M**	**F**	**M**		
**MBSQR**						
AE—Appearance evaluation	1.94	1.81	0.72	0.73	0.84	0.404
FE—Fitness evaluation	2.20	2.16	0.77	0.85	0.27	0.791
HE—Health evaluation	2.95	2.78	0.64	0.75	1.16	0.248
**AO—Appearance orientation**	3.47	3.11	0.67	0.64	2.43	0.017
FO—Fitness orientation	2.80	2.68	0.49	0.59	1.09	0.277
HO—Health orientation	3.29	3.10	0.51	0.46	1.74	0.086
IO—Illness orientation	3.09	3.24	0.84	0.65	−0.87	0.387
OWP—Overweight preoccupation	3.62	3.32	0.75	0.73	1.78	0.078
SCW—Self classified weight	4.81	4.56	0.41	0.98	1.81	0.073
BAS—Body areas satisfaction	2.21	2.32	0.55	0.56	−0.93	0.357
**EAT-26**						
B—Bulimia and food preoccupation	2.04	2.26	2.70	3.01	−0.35	0.729
EAT-B1 (overeating)	1.90	2.07	1.26	1.33	−0.59	0.556
EAT-B2 (use of laxatives)	1.04	1.07	0.20	0.38	−0.56	0.578
EAT-B3 (vomiting)	1.63	1.56	1.35	1.22	0.25	0.802
EAT-B4 (exercising more than 60 min a day)	1.64	2.19	1.05	1.59	−1.98	0.051

The comparison of the values of binary variables relating to “yes” or “no” answers to the following questions, namely “B5: Have you lost 10 kg or more of weight in the past 6 months?” and “B6: Have you ever been treated for an eating disorder?” was conducted using the Chi^2^ test (Table 3).

**Table 3:** Differences between men and women in terms of the frequency of responses given among the binary variables: EAT-B5 and EAT-B6.

**Variables**	**Response**	**F**		**M**	
		***n***	**%**	***n***	**%**
EAT-B5	Yes	27	36.99	6	22.22
${\mathrm{chi}}_{\left(1\right)}^{2}$ = 1.94; *p* = 0.163	No	46	63.01	21	77.78
EAT-B6	Yes	18	24.66	8	29.63
${\mathrm{chi}}_{\left(1\right)}^{2}$ = 0.25; *p* = 0.615	No	55	75.34	19	70.37

There were no statistically significant differences in the frequency of answering “yes” or “no” between men and women for B5 and B6. In the next step, a correlation analysis was conducted between the variables of body image and bulimic eating behavior (Table 4).

**Table 4:** Analysis of the correlation of predictors and dependent variables.

**Psychological variables**	**Bulimia and food preoccupation**	**EAT-B1 (overeating)**	**EAT-B2 (laxatives)**	**EAT-B3 (vomiting)**	**EAT-B4 (exercising more than 60 min)**
AE—Appearance evaluation	−0.34^**^	−0.21*	−0.01	−0.18	−0.06
FE—Fitness evaluation	−0.14	−0.01	0.02	−0.21^*^	0.05
HE—Health evaluation	−0.14	−0.09	0.25^*^	−0.09	−0.02
AO—Appearance orientation	−0.04	−0.02	0.17	0.10	−0.06
FO—Fitness orientation	−0.15	−0.15	−0.07	−0.03	0.22^*^
HO—Health orientation	0.01	−0.13	0.08	0.06	0.13
IO—Illness orientation	0.13	−0.05	0.18	0.05	0.02
OP—Overweight preoccupation	0.20^*^	−0.01	0.03	0.25^*^	0.09
SCW—Self classified weight	0.13	0.09	−0.04	0.01	0.01
BAS—Body areas satisfaction	−0.19	−0.16	0.17	−0.07	0.05

* *p < 0.05;*

** *p < 0.01*.

The dimensions of body image and dietary attitudes are correlated. The severity of the Bulimia dimension was associated with two body image scales: negatively with AE (*r* = −0.34) and positively with OP (*r* = 0.20). In terms of the dimensions in the second part of the questionnaire, B1 (overeating) was negatively correlated with variable AE (*r* = −0.21), Variable B2 (laxative use) was positively correlated with variable HE (*r* = 0.25). Variable B3 (vomiting) was negatively correlated with FE (*r* = −0.21) and positively with OP (*r* = 0.25). Variable B4 (exercising for more than 60 min per day) was positively associated with FO (*r* = 0.22). BMI severity was correlated poorly with HE (*r* = −0.20) and AO (*r* = −0.22).

The stepwise multiple and logistic regression analyses were used to answer the second question on the predictive role of body image dimensions for bulimic eating behavior in the study group. In addition to psychological predictors, BMI and the binary variables, namely and sex and age were included in the analyses. Table 5 presents the stepwise multiple regression analyses across the group.

**Table 5:** Body image dimensions as predictors of eating behavior in the whole group.

**Dependent variable**	**Predictors**	***B***	***Beta***	***r_**semi**_***	**% of variance**	***t***	***p***
Bulimia and food preoccupation	Constant	6.98				6.08	<0.001
*R* = 0.42; *R*^2^ = 0.18	AE	−1.12	−0.29	−0.28	8.03	−3.08	0.003
*F* = 10.38; *p* < 0.001	AGE	−0.07	−0.25	−0.25	6.04	−2.67	0.009
EAT-B1 (overeating) *F* = 1.14; *p* < 0.335	–	–	–	–	–	–	–
EAT-B2 (use of laxatives)	Constant	0.43				2.35	0.021
*R* = 0.33; *R*^2^ = 0.11	HE	0.11	0.29	0.28	8.10	2.97	0.004
*F* = 5.92; *p* < 0.004	AO	0.09	0.22	0.22	4.78	2.28	0.025
EAT-B3 (vomiting)	Constant	0.07				0.11	0.910
*R* = 0.25; *R*^2^ = 0.06	OP	0.43	0.25	0.25	6.25	2.56	0.012
*F* = 6.53; *p* < 0.012							
EAT-B4 (exercising more than 60 min a day)	Constant	−0.59				−0.77	0.442
*R* = 0.31; *R*^2^ = 0.10	FO	0.58	0.24	0.24	5.86	2.51	0.014
*F* = 5.21; *p* < 0.007	Sex	0.62	0.22	0.22	4.90	2.29	0.024

*R, multiple correlation coefficient; R^2^, multiple determination coefficient; F, F statistics for the analysis of variance for the whole model; p (at F), p probability for the model as a whole; B, partial regression coefficient; Beta, standardized partial regression coefficient; r_semi_, semi-particle correlation coefficient; % variance, the percentage of the variance of the dependent variable explained by the predictor (calculated as the square of the semi-partial correlation multiplied by 100%); t, value of the Student's t-statistics for the given predictor; p, p probability for a given predictor*.

For the Bulimia and food preoccupation variable, the model indicated a negative effect of two predictors, namely AE and Age (*R* = 0.42; *p* < 0.001), explaining 8.03 and 6.04% of the variance. While assessing the severity of behavioral variables in the preceding 6 months, no predictive model was matched for variable B1 (overeating). In regression models for dependent variables B2 (laxative use), B3 (vomiting) and B4 (exercising more than 60 min a day), the variables correlated positively. For variable B2, HE and AO proved to be predictors (*R* = 0.33, *p* = 0.004), explaining 8.1 and 4.78% of the variance. OWP was a predictor for B3 (*R* = 0.25; *p* = 0.012), explaining 6.25% of the variance. The model match (*R* = 0.31; *p* = 0.007) indicated that B4 could be explained by FO and sex, explaining 5.86 and 4.90% of the variance, respectively.

Table 6 shows the multiple stepwise logistic regression for the EAT-B5 and EAT-B6 binary variables. The analysis indicated that Nagelkerke's *R*^2^-value was 0.16 in the regression models for EAT-B5 and EAT-B6. The statistically significant predictors in multiple stepwise logistic regression for EAT-B5 (losing more than 10 kg in the past 6 months) were OWP and BAS. Both were positive, that is, as they increased, the probability of EAT-B5 increased (the odds ratios were 2.08 and 3.63, respectively). For EAT-B6 (treatment for eating disorders in the past), the predictor turned out to be HO (odds ratio 4.43), meaning that as it increased, the probability of EAT-B6 increased.

**Table 6:** Body image dimensions as predictors of eating behavior for EAT-B5 and EAT-B6 binary variables in the whole group.

**Predictors**	***B***	**SE**	**Wald**	***P***	**OR**	**95% CI for OR**	
**EAT-B5**							
OWP—Overweight preoccupation	0.73	0.33	4.89	0.027	2.08	1.09	3.98
BAS—Body areas satisfaction	1.29	0.45	8.34	0.004	3.63	1.51	8.72
Constant	−6.28	1.85	11.56	0.001	0.00		
**EAT-B6**							
HO—Health orientation	1.49	0.54	7.59	0.006	4.43	1.54	12.78
Constant	−2.90	2.23	1.69	0.194	0.05		

Table 7 presents the results of the stepwise multiple regression analysis for the female group. For the variable Bulimia and Food Preoccupation, the model (*R* = 0.30; *p* < 0.001) indicated an inhibitory role of AE, explaining 9.15% of the variance. As in the whole group, no regression analysis model was matched for B1, whereas positive correlations with explanatory variables were obtained for B2, B3, and B4. For B2 in the matched model (*R* = 0.34; *p* = 0.013), HO and BMI were predictive, explaining 6.91 and 5.11% of the variance, respectively.

**Table 7:** Body image dimensions as predictors of eating behavior in a group of women.

**Dependent variable**	**Predictors**	***B***	***Beta***	***r_**semi**_***	**% of variance**	***t***	***P***
Bulimia and food preoccupation	Constant	4.25				4.83	<0.001
*R* = 30; *R*^2^ = 09 *F* = 7.15; *p* < 009	AE	−1.14	−0.30	−0.30	9.15	−2.67	0.009
EAT-B1 (overeating)	–	–	–	–	–	–	–
*F* = 64; *p* < 797							
EAT-B2 (use of laxatives)	Constant	0.37				1.69	0.096
*R* = 34; *R*^2^ = 12	HO	0.10	0.26	0.26	6.91	2.34	0.022
*F* = 4.65; *p* < 013	BMI	0.01	0.23	0.23	5.11	2.01	0.048
EAT-B3 (vomiting)	Constant	−0.01				−0.01	0.991
*R* = 25; *R*^2^ = 06	OP	0.45	0.25	0.25	6.33	2.19	0.032
*F* = 4.80; *p* < 032							
EAT-B4 (exercising more than 60 min a day)	Constant	0.25				0.37	0.715
*R* = 23; *R*^2^ = 05	FO	0.50	0.23	0.23	5.48	2.03	0.046
*F* = 4.12; *p* < 046							

*R, multiple correlation coefficient; R^2^, multiple determination coefficient; F, F statistics for the analysis of variance for the whole model; p (at F), p probability for the model as a whole; B, partial regression coefficient; Beta, standardized partial regression coefficient; r_semi_, semi-particle correlation coefficient; % variance, the percentage of the variance of the dependent variable explained by the predictor (calculated as the square of the semi-partial correlation multiplied by 100%); t, value of the Student's t for the given predictor; p, p probability for a given predictor*.

The stepwise regression analysis on B3 (*R* = 0.25, *p* = 0.032) demonstrated the influence of the explanatory variable OWP with a coefficient of 6.33% of the variance. The last of the B4 variables—model match (*R* = 0.23; *p* = 0.046) indicated the influence of FO, explaining 5.48% of the variance.

Table 8 shows the multiple stepwise logistic regression for EAT-B5 and EAT-B6 in the female group. In the regression model for EAT-B5, Nagelkerke's *R*^2^-value was 0.13. BAS was found to be a statistically significant predictor in multiple stepwise logistic regression for EAT-B5 (losing more than 10 kg in the preceding 6 months). This means that as they increased, the probability of EAT-B5 increased (odds ratio: 9.70). In the regression model for EAT-B6 variable (past treatment for eating disorders), Nagelkerke's *R*^2^-value was 0.22. For this variable, the predictor turned out to be HO (odds ratio: 37.47), which means that as it increased, the probability of EAT-B6 increased.

**Table 8:** Body image dimensions as predictors of eating behavior for EAT-B5 and EAT-B6 for women.

**Predictors**	***B***	**SE**	**Wald**	***p***	**OR**	**95% CI for OR**	
**EAT-B5**							
BAS—Body areas satisfaction	1.28	0.51	6.42	0.011	3.60	1.34	9.70
Constant	−3.40	1.17	8.45	0.004	0.03		
**EAT-B6**							
HO—Health orientation	2.19	0.73	8.93	0.003	8.92	2.12	37.47
Constant	−8.60	2.59	11.00	0.001	0.00		

## Discussion

Body image and bulimic eating behavior are associated with many aspects that are important in the process of qualification for bariatric surgery and in the evaluation of the effectiveness of this form of treatment for people with obesity (32, 37). Studies confirm body dissatisfaction among obese individuals both before and after bariatric surgery, and in a longitudinal study model, it was found that levels of body image dissatisfaction decreased after surgery (25, 31, 43). Body dissatisfaction depends on BMI (43), but weight loss after surgery is not sufficient to reduce psychological stress, which is associated with body image satisfaction and increased emotional eating (31). Meneguzzo et al. showed that patients who underwent bariatric surgery encountered more difficulties after undergoing surgery in assessing their body size with respect to BMI than overweight and obese people who did not undergo surgery (44). Given the validity of the body image construct and bulimic behavior, we thought it would be interesting to establish the predictive role of multidimensional body image in bulimic-type eating behavior among individuals scheduled for the surgical treatment of obesity. The authors are aware of the importance of longitudinal studies. This research is a part of an on-going longitudinal study model designed to monitor patients after bariatric surgery.

While attempting to answer the first research question regarding differences in body image between men and women with obesity, differences were noted only in the AO dimension, which refers to caring about one's appearance and taking actions aimed at improving one's attractiveness. The difference was found to be significantly more intense in the female group. The result obtained is consistent with empirical data that confirm that women, not only among those with obesity but also in general populations, attach greater importance to the appearance and shape of their own bodies regardless of age and across cultural groups than do men (45–48). In the remaining body image dimensions and in the AE scale associated with the construct of the general body dissatisfaction index, there were no significant differences between the female and male subjects. Women and men did not differ in terms of the severity of their bulimic behavior. In contrast, differences emerged in the predictive role of sex for exercise performed with a frequency of more than 1 h per day in the preceding 6 months (B4 EAT-26) indicating that men are more physically active before bariatric surgery. Duncan et al. (49) found that distorted body image, greater preoccupation with weight and body shape, and eating patterns that are more prevalent among women than men may negatively affect the uptake of healthy lifestyle activities such as physical exercise. Heinberg et al. (50) indicated that people with moderate levels of body image dissatisfaction were more likely to engage in changing their eating patterns and introducing physical activity, with better results in weight reduction and improved well-being.

Preparing for bariatric surgery includes making lifestyle changes not only in relation to nutrition but also in implementing other health-promoting behaviors. Originally, in the EAT-26 test, high intensity values of this variable were identified with restrictive behavior; however, in the case of the group studied, this is an incorrect interpretation as physical activity was indicated and considered important in the process of treating obesity (51). Patients who increased their physical activity in preparation for surgery rated the improvement in the quality of life after surgery higher when compared to those who did not change their level of physical activity in the preoperative period (52).

The second research question was concerned with the determinants of bulimic behavior, understood as body image dimensions, BMI, age, and sex in the study group of patients qualified for bariatric surgery.

A strength analysis of the associations between indicators of multidimensional body image and bulimic eating behavior confirmed the relationships between these variables. Greater bulimic behavior was associated with greater dissatisfaction with one's appearance (AE) and higher levels of fear of weight gain. Stepwise multiple regression analysis indicated that AE and age were significant predictors for bulimic behavior across the group, but that age was not significant in the female group. This implies that body satisfaction and self-perceived attractiveness is a preventive factor in the occurrence of disordered eating behaviors, and that compulsive behaviors were more prevalent among people who were dissatisfied with their bodies. These results are consistent with empirical data presented in other studies in non-clinical (53) and clinical groups (37). Grilo et al. (37) indicated that body dissatisfaction, overvaluation of weight, preoccupation with weight, and fear of weight gain in a group of obese or overweight individuals with bulimic features or features of BED had significant effects on both the form of eating behavior and the frequency of its occurrence.

Preoccupation with weight and fear of weight gain (OP) significantly influenced eating behavior and was associated with vomiting for weight regulation (B3-EAT-26), both in the entire group and women's group alone. Similar weight control behaviors associated with laxative use (B2-EAT-26) were found to be conditioned in a sample of bariatric patients (female and male) by body image dimensions related to HE and AO. Those who rated their health better and placed greater importance on their physical appearance were more likely to engage in laxative behavior. Laxation was differently conditioned only in the group of women in whom a higher BMI and a higher level of HO, requiring adherence to a healthy lifestyle, induced laxative behavior. BMI severity was very weakly negatively associated with HE and AO, indicating that the more obese one is, the worse one feels and the less interest one has in their physical attractiveness. This result was consistent with studies that indicated that, compared to individuals with normal BMI, those with high BMI engaged in less health beneficial lifestyle behaviors (54). The severity of body dissatisfaction increased along with increasing BMI (43). Grilo et al. (37) studied groups of patients who were qualified for bariatric surgery (in a clinical sample that was similar to ours: predominance of women in the whole group, age, and BMI), and confirmed that as BMI decreases, satisfaction with body image increases. Geller et al. (31) showed that weight loss, however, is not sufficient after surgery to reduce psychological stress, which is related to body image satisfaction.

HO was a predictor of motivation to seek treatment for eating disorders. A quarter of the respondents indicated that they had been treated for this in the past. The analysis of the responses to this question did not differentiate between men and women. However, this result must be interpreted with caution, as there were respondents in this group who understood this question as referring to conservative treatment undertaken for obesity, rather than treatment undertaken for diagnosed, psychological eating disorders. This issue requires further research.

Weight preoccupation and fear of weight gain (OP) were significant predictors of behaviors related to weight loss by over 10 kg in the preceding 6 months (B5-EAT-26), but only in the general group. The effect of weight loss was determined by BAS in both the general group and the women's group alone. In our group, a third of the patients had reduced their weight by 10 kg or more in the preceding 6 months. However, patients qualified for bariatric surgery are advised to reduce between 5 and 10% of their initial body weight (55, 56), which, in clinical practice, means that some patients meet this criterion with a weight loss of <10 kg. These data can therefore be interpreted as favorable behavioral indicators of preparedness for surgical interventions and patient cooperation in treatments provided by multidisciplinary teams. Responses to the question on reducing 10 kg or more did not differ by sex. A weight reduction of 10 kg or more in the period before bariatric surgery was confirmed by 36.99% of women and 22.22% of men. Exercising for more than 60 min per day (B4-EAT-26) occurred at a higher frequency among men than among women, but these behaviors were also conditioned by the body image dimension of FO, that is, taking care of physical fitness as an important part of one's lifestyle. Both preoperative weight reduction through lifestyle changes (B5-EAT-26) and physical activity (B4-EAT-26) were desirable health-promoting strategies in the study group. Individuals are more likely to engage in changing their eating patterns and introduce physical activity, with better outcomes in weight reduction and improved well-being with moderate levels of body image dissatisfaction (50). Our study shows that these lifestyle behaviors (weight reduction and physical activity) are influenced by AE, OP, and FO.

The data obtained imply the importance of clinical assessment of body image dimensions in the context of qualification for bariatric surgery. The emergence of a group of patients with greater body dissatisfaction and excessive preoccupation with weight highlights the need for deeper psychological assessment for the presence of disordered eating patterns of the bulimic type. The identification of body image disorders and bulimic eating behaviors enables the application of specialized therapeutic procedures in preparation of a patient for bariatric surgery. The inclusion of the perspective of experiencing the body and its relationship with self-regulatory mechanisms related to eating is a valuable direction in psychological interactions, both before and after bariatric surgery (44, 57, 58). This may lead to better results in the surgical treatment of obesity, understood as the number of kilograms reduced in the long term, a reduction in unhealthy behaviors, and an improvement in the overall quality of life (59).

### Limitations of the Study and Prospects

This study used self-report methods that are limited by the risk of biased responses from the subjects. The small sample of men did not allow an assessment of the relationship in this group. The project is ongoing and was designed as a longitudinal study to assess the influence of psychological factors on surgical outcomes. We continue to recruit participants to join the study group from among patients qualified for bariatric surgery and seek to monitor patients after surgery at the Centre for the Surgical Treatment of Obesity at the University Hospital in Krakow.

## Conclusions

To contribute to the development of knowledge on the determinants of bulimic behavior and its relevance to the surgical treatment of obese patients, we focused on the determinants of these behaviors by analyzing multidimensional body image at the time of qualification for bariatric surgery. Despite the small number of men in the group, a clear difference between men and women emerged both in the context of body image and in the predictive role of sex in eating behavior. Our results showed that body image dimensions play a predictive role in various bulimic-type behaviors. On the one hand, AE, HE, OP, and AO are predictors for bulimic behaviors, whereas FO, BAS, and OP determine the treatment-promoting behaviors in a group of obese people. The results support the position that the patients' awareness of their body is important for the doctors to assess their psychological condition in the course of qualifying them for bariatric surgery. This is owing to the fact that some dimensions of body image are predictors of behaviors that serve as a risk factor for obesity and poor outcomes of bariatric treatment, whereas other dimensions increase the chances of pro-health behaviors among obese people.

## Data Availability Statement

The raw data supporting the conclusions of this article will be made available by the authors, without undue reservation.

## Author Contributions

B-BK and BI designed the study. AĆ and B-BK drafted application to the bioethical committee from the Jagiellonian University Medical College with the contribution of PM. PM qualified patients for bariatric surgery. AĆ qualified patients for the group and conducted psychological tests, conducted literature searches and provided summaries of previous research studies, and wrote the first draft of the sections Introduction and Discussion. B-BK conducted, analyzed and interpreted the data, and drafted the grant proposals that secured financing for the publication. B-BK, BI, and A-SF revised the manuscript. A-SF prepared the text to be placed in the Frontiers system. All authors contributed to the article and approved the submitted version.

## Funding

Open access license and publishing fee of this publication was funded by the Priority Research Area Society of the Future under the program Excellence Initiative—Research University at the Jagiellonian University in Krakow, Poland.

## Conflict of Interest

The authors declare that the research was conducted in the absence of any commercial or financial relationships that could be construed as a potential conflict of interest.

## Publisher's Note

All claims expressed in this article are solely those of the authors and do not necessarily represent those of their affiliated organizations, or those of the publisher, the editors and the reviewers. Any product that may be evaluated in this article, or claim that may be made by its manufacturer, is not guaranteed or endorsed by the publisher.
